# Mental health, quality of life and social relations in young adults born with low birth weight

**DOI:** 10.1186/1477-7525-10-146

**Published:** 2012-12-05

**Authors:** Line K Lund, Torstein Vik, Stian Lydersen, Gro CC Løhaugen, Jon Skranes, Ann-Mari Brubakk, Marit S Indredavik

**Affiliations:** 1 Regional Centre for Child and Adolescent Mental Health, Norwegian University of Science and Technology, Trondheim, Norway; 2 Department of Laboratory Medicine, Children’s and Women’s Health, Norwegian University of Science and Technology, Trondheim, Norway; 3 Department of Child and Adolescent Psychiatry, St. Olav’s University Hospital, Trondheim, Norway; 4 Department of Paediatrics and Rehabilitation, Sørlandet Hospital, Arendal, Norway; 5 Department of Paediatrics, St. Olav’s University Hospital, Trondheim, Norway

**Keywords:** Mental health, Quality of life, Social interaction, Very low birth weight, Small for gestational age, Young adult

## Abstract

**Background:**

Being born with low birth weight may have an impact on different aspects of mental health, psychosocial functioning and well-being; however results from studies in young adulthood have so far yielded mixed findings. The aim of this study was to assess the long-term impact in young adulthood on self-reported mental health, health-related quality of life, self-esteem and social relations by investigating differences between two low birth weight groups and a control group.

**Methods:**

In a follow-up at 20 years of age, 43 preterm VLBW (birth weight ≤ 1500 g), 55 term SGA (birth weight < 10th percentile) and 74 control subjects completed the Adult Self-Report (ASR) of the Achenbach System of Empirically Based Assessment, the Adult Autism Spectrum Quotient (AQ), the Short Form 36 Health Survey, the Self-Perception Profile for Adolescents-Revised, and the Wechsler Adult Intelligent Scale III assessment.

**Results:**

The VLBW and SGA groups reported significantly more mental health problems than controls. The VLBW group predominantly had internalizing problems, and the non-significant association with ASR Total score was reduced by the Intelligence Quotient (IQ). The SGA group had increased scores on both internalizing and externalizing problems, and the association with ASR Total score remained significant after adjusting for IQ in this group. Both low birth weight groups reported less interaction with friends and lower quality of life related to mental health domains than controls. Self-esteem scores were lower than in the control group for athletic competence (VLBW) and social acceptance (SGA).

**Conclusion:**

Our findings suggest that self-reported mental health and well-being in young adulthood may be adversely affected by low birth weight, irrespective of whether this is the result of premature birth or being born SGA at term.

## Background

There is emerging evidence that young adults born at very low birth weight (VLBW, birth weight: ≤ 1500 g) or extremely low birth weight (ELBW, birth weight < 1000 g) have an increased risk of emotional and behavioral problems [1-3]. According to findings from a large register based study, increased risk for hospitalization for a wide range of mental disorders was associated with preterm birth [4], and risk was inversely associated with gestational age. Others have reported increased risk for symptoms of depression [5], and problems related to ADHD [6] in VLBW young adults being born with signs of intrauterine growth restriction. Fetal growth restriction has also been linked to a possible increased risk of mental health problems in adolescents and young adults born at later gestational ages as well as at term, including depression and anxiety, but findings have been less conclusive [7-11]. In a previous paper, we reported an increased risk of psychiatric disorders among young adults born preterm at VLBW or who were born SGA at term (birth weight < 10th percentile adjusted for gestational age (GA), sex and parity) [12]. Furthermore, psychiatric morbidity in preterm born children has been associated with reduced cognitive functioning, as measured by intellectual quotient (IQ) [13].

Relational problems and autism spectrum traits have also been reported among children born preterm [13]. Higher rates of autism spectrum disorders have also been reported in late adolescence and adulthood, however, research is still scarce [14,15]. In a Finnish study on a VLBW young adult population, the VLBW group lagged behind the term born comparison group in establishing an adult independent life, including leaving the parental home and establishing intimate and cohabitant relationships [16]. According to a review by Hack, characteristics of adult social relations in preterm populations have so far yielded mixed findings [17].

Measures of quality of life (QoL) and self-esteem may illustrate a more overall impact on well-being. Most studies on QoL in various low birth weight populations have reported outcomes in young adulthood comparable to those of reference groups [18-23], although one study reported a moderately lowered objective QoL among VLBW individuals [24]. In a large follow-up study, young adults born SGA at term did not differ from controls in life satisfaction [25]. Studies on self-esteem in low birth weight populations in adulthood have so far generated inconsistent results [1,20,21,26,27].

Although some studies have focused on different aspects of mental health and well-being, few have addressed these questions in a comprehensive way. Therefore, in this study we wanted to explore self-reported mental health, QoL, self-esteem and social relations in two groups of young adults born with low birth weight, compared with a control group. Based on existing literature, we hypothesized that low birth weight individuals would report more mental health problems, similar health-related QoL, lower self-esteem and less contact with peers, compared with controls. We also hypothesized that IQ might influence mental health problems in the low birth weight groups compared with controls.

## Material and methods

### Study design

This is a population-based follow-up study of two low birth weight groups and a control group, all born between 1986 and 1988. One group was born preterm with very low birth weight (VLBW, birth weight: ≤ 1500 g) and the other was born small for gestational age (SGA) at term (birth weight < 10th percentile adjusted for gestational age (GA), sex and parity). The controls were born at term (birth weight ≥ 10th percentile, adjusted for GA, sex and parity).

During the enrolment period, all VLBW participants were admitted to the referral neonatal intensive care unit for the counties of North and South Trøndelag at The University Hospital in Trondheim, Norway. In the same period, the SGA and control participants were enrolled as part of a multicenter study comprising participants from Uppsala, Sweden, and Trondheim and Bergen, Norway [28]. A 10% random sample of women was selected for follow-up during pregnancy. At birth, all children of mothers in the random sample and all children born SGA in the nonrandom sample were included for follow-up. Of these, only SGA and control participants recruited in Trondheim were included in the present study. Data were collected between autumn 2006 and autumn 2008.

### Study population

#### The preterm VLBW Group

During 1986–88, 99 VLBW children were admitted to the neonatal intensive care unit, of whom 23 died and one with a congenital syndrome was excluded. At follow-up, additionally two were excluded because of severe cerebral palsy (CP) and/or mental retardation. Of the 73 eligible, 14 could not be traced. Hence, 59 were invited to participate, of whom 43 (17 men, 26 women) consented to participate in this part of the study (73% of 59 invited and 59% of 73 eligible). Eleven (26%) were born SGA (VLBW–SGA). The reference standards for classification of SGA (below 10th percentile) were specific for each sex and gestational week based on data from the Norwegian Medical Birth Registry [29]. Group characteristics were mean (SD) birth weight: 1236 g (205), gestational age: 29.0 (2.5) weeks, assessment age: 19.5 (0.6) years and parental socioeconomic status (SES, *n* = 41): 3.3 (1.4).There were no significant differences compared with controls in assessment age or parental SES. Two VLBW participants had CP (both bilateral spastic subtype, one of these with four limbs affected).

#### The term SGA Group

Among eligible women in the Trondheim part of the multicenter study, 104 of the 1200 (9%) gave birth to an SGA child at term. One newborn with a congenital syndrome was excluded. Of 103 eligible, 17 had moved or could not be traced. Hence, 86 were invited to participate, of whom 55 (24 men, 31 women) consented to participate in this part of the study (64% of 86 invited and 53% of 103 eligible participants). Group characteristics were mean (SD) birth weight: 2911 g (240), gestational age: 39.6 (1.2) weeks, assessment age: 19.8 (0.7) years and mean parental SES (*n* = 47): 3.5 (1.2). There were no significant differences compared with the control group in gestational age, assessment age or parental SES. One participant had CP (spastic bilateral subtype).

#### The control group

This group comprised 120 children recruited from the random sample. Two with a congenital syndrome were excluded. Of 118 eligible participants, 16 had moved or could not be traced. Hence, 74 of 102 invited subjects (31 men and 43 women) participated in this part of the study (73% of 102 invited and 63% of 118 eligible). Group characteristics were mean (SD) birth weight: 3716 g (473), gestational age: 39.8 (1.2) weeks, mean age at assessment: 19.7 (0.5) years and parental SES (*n* = 67): 3.6 (1.0).

#### Nonparticipants

There were no statistically significant differences in birth weight, gestational age and head circumference at birth between participants and those who did not consent to participate (data not shown). In the VLBW group, the proportion of men was higher among those who declined to participate (12/16 (75%) compared with those who participated (17/43 (40%), *p* = 0.015).

### Outcome measures

Self-reported mental health was obtained by the Achenbach System of Empirically Based Assessment (ASEBA), Adult Self-Report (ASR, age range from 18–59) [30], which comprises 123 problem items rated as “not true” (0), “somewhat or sometimes true” (1), or “very true or often true” (2). Co-occurring problems are clustered into the following Syndrome Profiles according to the manual: Anxious/Depressed, Withdrawn, Somatic Complaints (comprising the composite scale for Internalizing Problems), Aggressive Behavior, Rule-Breaking Behavior and Intrusive Behavior (comprising the Externalizing Problems composite scale), Thought Problems and Attention Problems. The Total Problems score is the sum of all problem items. Higher scores indicate more problems.

The Adult Autism Spectrum Quotient (AQ) (self-report) [31] was used to assess traits within the autistic spectrum. The AQ consists of 50 items, scored 0 or 1, grouped into five different domains: social skills, attention switching, attention to detail, communication and imagination. The maximum total score is 50; higher scores indicate more autistic traits. A clinical cutoff value is set at a total AQ score ≥ 32 [31]. A Norwegian pilot version was used with permission from the original author. We (the first author and a colleague) have since slightly revised and translated the AQ according to standard procedure, and this version is available at http://www.autismresearchcentre.com. This questionnaire presumes normal intelligence. Analyses were performed only on those participants who had also been tested cognitively. As we wanted to explore results for our whole sample, scores are given both including and excluding those with cognitive disability (defined as IQ < 2 SD of mean IQ score) in the control group.

Health-related QoL was evaluated by the Short Form 36 (SF-36). This questionnaire comprises 36 statements summarized in eight transformed subscales, ranging from 1–100 [32]: mental health (5 items), vitality (4 items), social functioning (2 items) and role limitations due to emotional problems (3 items), general health (5 items), physical functioning (10 items), bodily pain (2 items) and role limitations due to physical problems (4 items). Higher scores indicate favorable health outcomes. Psychometric properties have been evaluated in a Norwegian representative sample and Norwegian normative data are available [33].

Self-esteem was evaluated through the Self-Perception Profile for Adolescents-Revised (SPPA-R) [34]. SPPA-R consists of 35 statements rated from 1–4, divided into seven domain-specific subscales: close friends, school competence, social acceptance, athletic competence, physical appearance, romantic appeal and global self-worth. Higher scores indicate positive self-perceptions. The psychometric properties of SPPA-R have been evaluated in a Norwegian national representative sample for ages 13–19 years, and Norwegian norms are available [34]. Analyses were conducted with and without individuals with CP.

Information on relations to friends and family was obtained by ASR Adaptive scores. Questions on friends included number of close friends, frequency of contact with friends, getting along with friends, visits from friends, rated 0–3. A total score for relation to friends was calculated with a maximum score of 12, higher scores indicate better adaptive functioning. Information on family relations included the self-perceived relationship with parents and/or siblings compared with others, and was rated worse (0), average (1) or better (2). In addition, participants were asked study-specific questions about current housing and daily occupation.

The Wechsler Adult Intelligent Scale (WAIS III) [35] was administered by a trained neuropsychologist to measure the participants’ full-scale intelligence quotient (IQ) based on age-appropriate US norms. We obtained data on IQ for 41 VLBW, 53 SGA and 73 control participants. Both low birth weight groups had significantly lower IQ scores than controls; results are presented in the Additional file 1: Table S1.

Parents’ socioeconomic status (SES) was recorded according to Hollingshead’s two-factor index of social position rated from 1 (lowest) to 5 (highest) based on the parent’s education and occupation [36]. The SES score was calculated at 14 years of age and supplemented with data at 20 years. Information on SES was available for 41 VLBW, 47 SGA and 67 control participants.

### Ethics

The Regional Committee for Medical and Health Research Ethics in Central Norway approved the study protocol (Project number: 4.2005.2605). Participation was based on written informed consent. Participants in need of mental health care were offered referral to appropriate health services for further evaluation and assistance.

### Statistics

The groups were compared using one-way ANOVA and Scheffe’s post hoc test for normally distributed data and Kruskal–Wallis test and Mann–Whitney test for data not normally distributed. Comparisons of proportions were made by Pearson’s chi-squared test or the exact unconditional z-pooled test. Normality of the ASR variable was checked by visual inspection of Q–Q plots. Although ASR Total Problems score was not normally distributed, the square root transformed score was approximately normally distributed. This score was consequently used in linear regression analysis to compare the ASR Total Problems score in the low birth weight groups with the control group as reference. Potential confounders (assessment age, SES (parental) and sex) and IQ were each adjusted for separately.

Missing items were handled according to the manual for ASR and SF-36. In ASR, one subject in the VLBW group had a missing item on “friends visit” and ASR problem scores were not calculated for one subject in the VLBW and one in the control group because of missing items. On SF-36, one subject in the control group had too many missing items to be included. Data were missing on AQ for three subjects in the control group. Missing items were handled by simple mean imputation on AQ and SPPA-R. On AQ, subscales were only calculated if fewer than five items were missing in total [37] and if ≥ half of items in each subscale were completed. On the SPPA-R, subscales were only calculated if ≥ 80% was completed.

Cognitive disability was defined as IQ < 2 SD of the mean IQ score in the control group (i.e. IQ < 77.14). Cognitive disability was found in nine of 41 (22%) VLBW and four of 53 (8%) SGA participants, but in none of the control group.

We used SPSS version 17.0 (PASW statistics) (SPSS Inc., Chicago, IL) for data analysis. A two-sided p-value < 0.05 was considered significant and 95% confidence intervals are given when appropriate. Due to the large number of comparisons p-values between 0.01 and 0.05 should be interpreted with caution.

## Results

### Mental Health According to ASEBA, Adult Self-Report (ASR)

Mental health problems are shown in Table 1. The VLBW group had higher scores than controls on the syndrome scale for Anxious/Depressed and on the composite scale for Internalizing problems (p between 0.01 and 0.05). The SGA group had significantly higher scores than controls on the scales for Anxious/Depressed, Withdrawn, Thought Problems, Attention Problems, Aggressive Behavior, Internalizing Problems and Total Problems (p < 0.01), and for Somatic Complaints and Externalizing Problems (p between 0.01 and 0.05) .

**Table 1:** ASEBA Adult Self-Report in two groups of young adults born with low birth weight and a control group

	**VLBW**			**SGA**			**Control**	
	**Mean**	**(SD)**	***p***	**Mean**	**(SD)**	***p***	**Mean**	**(SD)**
*Problem scores* (*n* = 42/55/73)								
Anxious/Depressed	6.8	(7.0)	0.029	8.6	(7.6)	< 0.001	4.2	(5.1)
Withdrawn	2.0	(2.2)	0.22	3.1	(2.9)	< 0.001	1.5	(1.8)
Somatic Complaints	2.7	(2.8)	0.057	3.5	(4.3)	0.015	1.7	(2.3)
Thought Problems	2.1	(2.4)	0.16	2.9	(3.2)	< 0.001	1.3	(1.8)
Attention Problems	7.0	(4.9)	0.16	8.0	(4.7)	0.006	5.7	(4.2)
Aggressive Behavior	4.1	(3.8)	0.079	5.3	(5.2)	0.008	2.9	(3.1)
Rule-breaking Behavior	2.4	(2.6)	0.36	3.0	(3.3)	0.091	2.1	(2.6)
Intrusive	1.2	(1.2)	0.17	2.3	(2.1)	0.23	1.8	(1.8)
Internalizing Problems	11.6	(10.7)	0.042	15.1	(13.3)	< 0.001	7.4	(7.3)
Externalizing Problems	7.7	(6.1)	0.38	10.5	(9.0)	0.019	6.7	(5.9)
Total Problems	35.5	(24.4)	0.067	46.0	(30.2)	< 0.001	27.8	(20.8)
*Adaptive scores* (*n* = 42/55/74)								
Friends scale (Total score)	10.3	(1.7)	0.023	10.4	(1.6)	0.010	11.1	(1.1)
Family scale (Total score)	1.4	(0.5)	0.11	1.5	(0.4)	0.58	1.5	(0.4)

*P*-values vs. controls based on Mann–Whitney *U* test.

The effect of being born at VLBW on the square root-transformed Total Problems score was largely unchanged after adjusting for assessment age, SES and sex (Table 2). Adjusting for total IQ score attenuated the effect of being born at VLBW on the dependent variable. The effect of being born SGA on the dependent variable remained significant after adjusting for assessment age, SES, sex and IQ (Table 2). The interactions between sex and group scores were not significant (data not shown).

**Table 2:** Linear regression of the square root-transformed total problems score of the ASEBA Adult Self-Report as dependent variable in young adulthood in two groups born with low birth weight compared with a control group

	**VLBW**			**SGA**		
	**B**	**95% Confidence Interval**	***p***	**B**	**95% Confidence Interval**	***p***
Unadjusted*	0.7	(−0.1 to 1.5)	0.079	1.5	(0.8 to 2.3)	< 0.001
Adjusted for:						
Age (*n* = 42/55/73)	0.8	(−0.1 to 1.6)	0.065	1.5	(0.8 to 2.3)	< 0.001
SES (*n* = 40/47/66)	0.7	(−0.1 to 1.6)	0.097	1.3	(0.5 to 2.1)	0.002
Sex (*n* = 42/55/73)	0.7	(−0.1 to 1.5)	0.081	1.5	(0.8 to 2.3)	< 0.001
IQ (*n* = 40/53/72)	0.2	(−0.6 to 1.1)	0.59	1.4	(0.7 to 2.2)	< 0.001

*Unadjusted values with the control group as reference.

Unadjusted analysis including only those with calculated parental SES: VLBW: B: 0.7 (CI: –0.1 to 1.6), *p* = 0.091, SGA: B: 1.3 (CI: 0.5 to 2.1), *p* = 0.002.

### Autistic Traits According to the Adult Autism Spectrum Quotient (AQ)

The VLBW group had higher scores than controls on the subscales measuring social skills and attention switching, and the AQ total score was higher (Table 3). After excluding participants with cognitive disability, only scores on the attention switching subscale remained significantly higher than controls. SGA participants had higher scores on the subscales for social skills and attention switching, which was maintained when participants with cognitive disability were excluded. The total AQ score in the SGA group did not differ significantly from that in controls (Table 3). No participants in the low birth weight groups, but one in the control group, had AQ scores ≥ 32.

**Table 3:** The Adult Autism Spectrum Quotient (AQ) self-report in two groups of young adults born with low birth weight and a control group

	**(*n*** **= 40/53/70) a)**	**VLBW**			**SGA**			**Control**	
	**(*n*** **= 31/49/70) b)**	**Mean**	**(SD)**	***p***	**Mean**	**(SD)**	***p***	**Mean**	**(SD)**
Communication	a)	2.1	(1.3)	0.24	2.2	(1.9)	0.44	1.9	(1.6)
	b)	1.9	(1.2)	0.59	2.0	(1.8)	0.81		
Social skills	a)	1.7	(1.7)	0.013	1.7	(1.9)	0.012	1.0	(1.4)
	b)	1.4	(1.7)	0.28	1.6	(1.8)	0.036		
Attention switching	a)	4.6	(2.1)	0.005	4.6	(2.1)	0.003	3.4	(1.8)
	b)	4.5	(2.1)	0.023	4.6	(2.1)	0.005		
Attention to detail	a)	3.9	(2.0)	0.99	3.5	(1.8)	0.29	3.9	(1.9)
	b)	3.9	(1.9)	1.00	3.4	(1.7)	0.20		
Imagination	a)	3.0	(1.7)	0.69	3.0	(1.8)	0.83	2.9	(1.8)
	b)	2.7	(1.4)	0.70	2.9	(1.8)	0.96		
Total AQ score	a)	15.4	(4.6)	0.010	14.9	(6.1)	0.082	13.1	(5.1)
	b)	14.3	(4.2)	0.12	14.4	(6.0)	0.22		

*P*-values vs. controls based on Mann–Whitney *U* test.

a) Analyses including participants with cognitive disability, b) Analyses excluding participants with cognitive disability.

### Health-related QoL according to the short form 36 (SF-36)

Compared with controls, the VLBW group had lower scores on the scale for mental health (p between 0.01 and 0.05) and the SGA group had lower scores on the scales for mental health, social functioning and emotional role (p ≤ 0.01), (Table 4).

**Table 4:** Short Form 36 (SF-36) scores in young adulthood in two groups born with low birth weight compared with normal birth weight controls

	**VLBW (*n*** **= 43)**			**SGA (*n*** **= 55)**			**Control (*n*** **= 73)**	
	**Mean**	**(SD)**	***p***	**Mean**	**(SD)**	***p***	**Mean**	**(SD)**
Mental Health	73.6	(15.0)	0.028	70.0	(18.1)	0.001	79.2	(11.9)
Vitality	50.1	(19.1)	0.11	50.2	(21.6)	0.14	56.2	(14.2)
Social Functioning	91.0	(12.6)	0.25	81.6	(22.7)	0.001	92.6	(13.1)
Role–Emotional	87.6	(24.2)	0.22	70.3	(38.8)	<0.001	90.9	(23.7)
General Health	79.3	(17.8)	0.89	73.4	(24.2)	0.26	78.7	(19.8)
Physical Functioning	90.2	(20.4)	0.24	95.3	(7.1)	0.45	95.5	(10.1)
Bodily Pain	80.2	(22.6)	0.98	80.5	(23.5)	0.81	80.2	(22.5)
Role–Physical	89.0	(19.1)	0.18	83.2	(30.1)	0.077	91.1	(22.2)

*P*-values vs. controls based on Mann–Whitney *U* test.

### Self-esteem according to the Self-Perception Profile for Adolescents-Revised (SPPA-R)

The mean athletic competence score was lower for VLBW participants than controls (Table 5). SGA participants had a lower score than controls on the social acceptance scale. After excluding participants with CP, scores were largely unchanged (data not shown).

**Table 5:** Self-esteem in young adulthood in seven domain-specific areas according to Self-Perception Profile for Adolescents-Revised (SPPA-R) in two groups of low birth weight compared with normal birth weight controls

	**VLBW *n*** **= 43**			**SGA *n*** **= 55**			**Control *n*** **= 74**	
	**Mean**	**(SD)**	***p***	**Mean**	**(SD)**	***p***	**Mean**	**(SD)**
School competence	2.9*	(0.6)	0.35	2.8	(0.6)	0.051	3.0	(0.6)
Social acceptance	3.4	(0.5)	0.17	3.2	(0.7)	0.008	3.5	(0.4)
Athletic competence	2.3	(0.7)	< 0.001	2.6	(0.7)	0.12	2.8	(0.6)
Physical appearance	2.8	(0.8)	0.89	2.7	(0.8)	0.35	2.8	(0.7)
Romantic appeal	2.7	(0.6)	0.15	2.7	(0.6)	0.18	2.9	(0.5)
Close friends	3.4	(0.6)	0.088	3.4	(0.6)	0.12	3.6	(0.4)
Global self-worth	3.1	(0.8)	0.30	3.0	(0.8)	0.58	3.1	(0.5)

*P*-values vs. controls based on Mann–Whitney *U* test.

* One missing value (*n* = 42) in the VLBW group on the subscale of school competence.

### Relations with friends and family and daily occupation

According to the ASR Adaptive scores, the VLBW participants had fewer visits from friends or family (p = 0.04), spent less time with friends (p = 0.001) (data not shown), and had a lower mean friends total score than controls (Table 1). The SGA group had fewer visits from friends or family (p = 0.002, data not shown), and had a lower friends total score than controls. Both VLBW participants and SGA participants perceived their relations with parents and/or siblings as comparable to those of controls (Table 1).

Fewer VLBW and SGA participants than controls were students in higher educational institutions, employees, or in military service (Additional file 1: Table S1). There were no significant group differences in the proportions of subjects living with one or both parents (Additional file 1: Table S1). One VLBW and one control participant had children of their own.

## Discussion

In this study, young adults born preterm VLBW and term SGA reported more problems related to mental health, well-being and social relations than normal birth weight controls. The findings were evident across various questionnaires. IQ had an effect on mental health variables in the VLBW group, while in the SGA group the association with mental health problems was still highly significant after adjusting for IQ. This study highlights the long-term impact on mental health, social relations, QoL and self-esteem of being born at low birth weight, thereby extending former knowledge on this important topic.

### The VLBW Group

The VLBW participants had higher mean scores than controls on self-reported Anxious/Depressed and Internalizing Problems scales. These findings are in line with other studies [1-3] and with our previous report of a high prevalence of anxiety disorders in young adults in this group [12]. We also diagnosed more attention deficit hyperactivity disorder in the same study using in-depth psychiatric interview; however, in the present study there were no significant group differences compared with controls on the ASR attention subscale. Hence, ASEBA self-report revealed fewer problems in this group than expected from the in-depth interview. This discrepancy is consistent with our follow-up study in the same VLBW group at 14 years of age [38]. Furthermore, dissimilar ratings on subscales of ASEBA parent-reports and self-reports in VLBW young adult populations have been described [2,3]; hence non-significant group differences on self-reports should be carefully interpreted.

The higher ASR Total Problems score could not be explained by age at assessment, sex or parental SES, while the same score was reduced when adjusted for full-scale IQ in the VLBW group. Moreover, the higher total scores on autistic traits compared with controls became nonsignificant when participants with cognitive disability were excluded. We found that alterations in brain white matter were associated with cognitive measures and mental health variables among VLBW adolescents at 14 years of age [39]. We therefore speculate that in VLBW individuals, reduced intellectual capacity and comorbid impaired mental health may be largely explained by a deviant brain development persisting into adulthood.

Anxiety, inattention and social problems have been suggested to constitute a “preterm behavioral phenotype” [13]. Autistic traits may affect social skills. Risk of autism is associated with perinatal factors [40]. Increased prevalence of autism spectrum disorders and increased rates of disability pension related to autism spectrum disorders has been reported in adolescence and adult low birth weight populations [14,15]. In this study the VLBW group had more problems related to attention switching than controls on the AQ questionnaire. Social interaction demands compound skills, and the ability to keep track of simultaneous and complex information in a social setting may be affected by problems in attention switching [37]. Attention problems and cognitive disability may therefore contribute to social problems in the VLBW group, as previously suggested by others [41-43]. Less interaction with friends was reported on the ASR Adaptive score in this group, as has also been previously reported in young adult women born at VLBW [2]. Our findings of more anxiety, attention and subtle social problems may indicate that the “preterm behavioral phenotype” may also apply in young adulthood.

Despite expectations, but consistent with the results of the ASR, mental health domain of QoL was affected by being born at VLBW. This is in line with the negative association between internalizing problems and outcome in health related quality of life found among Dutch young adults born at VLBW or born very preterm [44]. On SPPA-R, the VLBW young adults reported lower self- esteem on athletic performance than controls. Others have demonstrated less physical activity among adolescents and young adults born at very low birth weight [20,45], and that men born very preterm reported lower physical functioning than full-term controls [26]. Except for lower scores in relation to mental health and athletic performance, other domains of QoL and self-esteem did not differ significantly from controls. One possible explanation may be that the VLBW young adults may have adapted to their situation [23,24].

### The SGA Group

In the SGA group, the problem scores were higher than in the control group for a wide range of mental health problems. The group differences overall were large and consistent with our previously reported finding of higher psychiatric morbidity compared with controls, based on diagnostic assessment [12]. Furthermore, the higher problem scores are in line with studies reporting an increased risk of mental health problems in populations of adults who suffered intrauterine growth restriction (IUGR) [7,10,46]. However the large difference in self-reported mental health problems between subjects born SGA and the controls in a cohort with relatively modest growth restriction is unexpected, keeping in mind that studies of mental health outcome in IUGR populations have so far produced ambiguous findings [8,9]. The higher scores compared with controls on mental health problems could not be explained by age at assessment, parental SES or sex. In contrast to the findings in the VLBW group, they were also not explained by a lower full-scale IQ. Thus, abnormal brain development is less likely to cause mental health problems in young adults born SGA at term. Instead, such problems support the hypothesis of a possible intrauterine programming associated with impaired fetal growth [46]. Intrauterine programming includes probable alterations of the endocrine Hypothalamus- Pituitary- Adrenal (HPA) stress-regulating system [47,48]. We therefore speculate that the high mental health problem scores reported by young adult SGA participants may be partly explained by a reduced tolerance to stress. This may also play a role in the reported low scores related to mental health domains of QoL and the social acceptance scale of self-esteem. Furthermore, health-related QoL in children and adolescents may decrease over time if mental health problems increase [49]. In a recent study of the same population, we found that mental health problems increased significantly in the SGA group between 14 and 20 years of age [50]. Hence, another possible interpretation of the low QoL measures in the SGA group may be increased mental health problems from adolescence to young adulthood.

### Mental health and related concepts

In sum, both groups of low birth weight reported psychological distress, problems with attention switching, affected self-esteem in some domains and reduced quality of life in relation to mental health. Indeed, the sub-scales of SF-36 related to mental health may reflect the higher problem scores reported on ASR. Lower quality of life is associated with mental health problems [21], and health related quality of life may decrease over time if mental health problems increase [49]. Furthermore, in general, self- esteem in adolescence may predict mental health problems in adulthood [51] and social relations may be negatively affected by mental health problems. Hence, there is reason to believe that quality of life, self-esteem, social relations and mental health problems are associated concepts, and more research is needed to detect how these problems are interrelated in low birth weight populations.

### Clinical and research implications

Our findings suggest that low birth weight may be a risk factor for adverse long term mental health outcome irrespective of whether the cause is preterm birth or intrauterine growth restriction. In this study, the impact of being born VLBW on self-reported mental health problems was reduced when adjusting for IQ. Consequently, psychiatric assessment should include a cognitive examination to facilitate prevention and treatment of mental health problems in this group. Furthermore, as discussed, the young VLBW adults reported less mental health problems on self-reported questionnaires than they did during psychiatric interview. Hence, supplementing self-reports with diagnostic assessment, and also information from multiple informants, may increase the validity of mental health assessment in this group. Young adults born SGA at term reported comprehensive mental health problems and reduced mental health domains of QoL. As this group constitutes a relatively large group of people, there is a potentially high attributable risk.

The findings on mental health in the VLBW group add and extend previous international research on prematurely born children and may probably be generalized to VLBW young adults born from the same geographically based year cohorts in Norway and other developed countries. However, due to significant improvements in perinatal care during the last three decades, caution is needed in generalizing our results to infants born with VLBW in more recent years. Thus, further research including continuous long term follow-up is needed to address the external validity of our findings. Our results in the SGA group represent new findings that need to be replicated in independent samples. Hence; in clinical practice as well as in research, continued attention on the long term mental health outcome and associated risk factors seems indicated.

It will be of interest to see if problems are transitional or if they continue as the subjects grow older. As many psychiatric disorders emerge during adolescence, further research on these low birth weight groups should address adolescent as well as perinatal factors that could possibly trigger adult psychopathology.

### Strengths and limitations

The strengths of this study are the longitudinal design into young adulthood and the inclusion of both a preterm and a term born group with low birth weight, by that highlighting some possible long term consequences of both prematurity and intrauterine growth restriction. Furthermore, in this study, we used a variety of measures, providing a broad description of how mental health and well-being were perceived by the young adults themselves.

The limited sample size made separate analyses for men and women difficult, and reduced the power to detect small differences between groups; hence negative findings should be interpreted warily. Although significant differences between the low birth weight groups and controls were found across various measures, the significant p-values in the VLBW group were mostly between 0.01 and 0.05 and should therefore be interpreted with greater caution than the overall highly significant p-values in the SGA group. On the other hand it may be emphasized that all findings were coherent, suggesting more mental problems and reduced well-being in both low birth weight groups, compared with controls. A possible selection bias cannot be excluded in the VLBW group, as the proportion of men was higher among those who declined to participate than in those who participated. Most mental disorders seem to be more frequently occurring in women than in men in the adult population [52]. As there is a greater preponderance of women in our study groups, an amplification of mental health problems and associated factors cannot be excluded. Since SGA is a statistically defined concept, this group comprises subjects born both with and without growth restriction. This misclassification is likely to dilute the real effect of being born with IUGR, and is therefore unlikely to explain the substantial differences between the SGA and the control group. The collected information is based solely on self-reports, but as the participants are adults, this is probably the way they would have represented themselves in most settings at this age. We used a pilot version of the AQ and slight changes made during the subsequent completion of the translation process may reduce the ability to replicate our results.

## Conclusions

This study shows that being born preterm with VLBW or SGA at term may have a long-term negative influence on mental health, QoL, self-esteem and social interaction in adulthood. The VLBW group had predominantly internalizing problems on self-report and mental health scores were reduced when adjusting for IQ in this group. The SGA group reported a wide range of mental health symptoms. In both low birth weight groups, mental health aspects of QoL were affected. Further research into adulthood of children born preterm or at term with IUGR is crucial to document the long-term impact of low birth weight. This may in turn guide initiatives to prevent and treat mental health problems and its impact on daily life.

## Abbreviations

VLBW: Very Low Birth Weight; SGA: Small for Gestational Age; GA: Gestational Age; IUGR: Intrauterine Growth Restriction; ASEBA: Achenbach System of Empirically Based Assessment; ASR: Adult Self- Report; AQ: Adult Autism Spectrum Quotient; SF-36: Short Form 36; QoL: Quality of Life; SPPA-R: Self-Perception Profile for Adolescents-Revised; WAIS: The Wechsler Adult Intelligent Scale; IQ: Intelligence Quotient; SES: Socioeconomic status; ADHD: Attention Deficit Hyperactivity Disorder; CI: Confidence Interval; ANOVA: Analysis Of Variance.

## Competing interests

The authors declare that they have no competing interests.

## Authors’ contributions

LKL, MD: Participated in the design, coordinated and collected data on mental health, quality of life, self- esteem and social relations, translated the AQ questionnaire, performed data analyses and drafted the manuscript. TV, MD PhD: Conceived of the study, participated in the design and coordination of the study and helped to draft the manuscript. SL, PhD: Supervised on and performed data analyses and helped to draft the manuscript. GCCL, Neuropsych PhD: Coordinated and collected data on cognitive variables and helped to draft the manuscript. JS, MD PhD: Participated in the design and coordination of the study and helped to draft the manuscript. AMB, MD PhD: Conceived of the study, participated in the design and coordination of the study and helped to draft the manuscript. MSI, MD PhD: Conceived of the study, participated in the design and coordination of the study and helped to draft the manuscript. All authors have read and approved the final manuscript.

## Supplementary Material

Additional file 1 **Table S1.**Daily occupation, current housing and full-scale IQ in young adulthood in two groups born with low birth weight compared with controls. Click here for file
