# Supplementary material for: Mental health, quality of life and social relations in young adults born with low birth weight
Source: Health Qual Life Outcomes. 2012 Dec 5;10:146. doi: 10.1186/1477-7525-10-146 (PMC3541130; doi:10.1186/1477-7525-10-146)
Supplement: Additional file 1 — Table S1.Daily occupation, current housing and full-scale IQ in young adulthood in two groups born with low birth weight compared with controls. [file 1477-7525-10-146-S1.doc]

**Supplementary Table** Daily occupation, current housing and full-scale IQ in young adulthood in two groups born with low birth weight compared with controls

|  | VLBW | | | SGA | | | Control |  |
| --- | --- | --- | --- | --- | --- | --- | --- | --- |
|  | *n* | (%) | *p* | *n* | (%) | *p* | *n* | (%) |
| Daily occupation, *n* = 43/55/74 |  |  |  |  |  |  |  |  |
| School^†^ | 24 | (56) | 0.020 | 25 | (45) | 0.132 | 25 | (34) |
| Other^‡^ | 13 | (30) | < 0.001 | 26 | (47) | 0.046 | 48 | (65) |
| No organized daily occupation | 6 | (14) | 0.005 | 4 | (7) | 0.097 | 1 | (1) |
| Current housing*, *n* = 42/55/74 |  |  |  |  |  |  |  |  |
| Proportion living with parents Yes/No^§^ | 28/14 | (67/33) | 0.082 | 34/21 | (62/38) | 0.182 | 37/37 | (50/50) |
|  | Mean | (SD) | *p* | Mean | (SD) | *p* | Mean | (SD) |
| Full-scale IQ, *n* = 41/53/73 | 89 | (12) | < 0.001 | 94 | (10) | 0.005 | 101 | (12) |

*P*-values vs. controls

* Missing information for one VLBW participant

^†^ Upper secondary school or nongranting college

^‡^ Employee, higher education or military service

^§^ No: Living alone, with roommates, married, in cohabitant relationships
